# Lives of Medical Men

**Published:** 1878-12

**Authors:** 


### LIVES OF MEDICAL MEN.

Died June 13th 1867. M. Civiale, the greatest Lithotritist of his time, aged seventy-five years; ten days later Died Trousseau, after months of cruel sufferings from cancer of the stomach; he was the first to determine what was the nature of his disease and the point which gave him most uneasiness was that he would be subject to the inconvenience of starving to death; but as the malady progressed, and he found that he had less and less appetite, he seemed quite reconciled to his fate and seemed to have nothing more to desire. Dr. Trousseau was the first in modern times to draw public attention to nitrate of silver as a cure for some throat diseases; soon after that, medical men of this country became hallucinated on the subject, and the moment a man seemed to be aware that he had a throat, by a tickling or clearing or cough or lumpy feeling there, which could be swallowed away, and you could feel it go down clear out of sight, and then at the very next swallow was ready to go down again, down went a swab, almost choking you to death, and in five minutes, two minutes, one minute, the tickling was gone, your cough was gone, your common sense was gone, and you jumped up out of your chair and in most exstatic mood declared to every man, woman and child met with in the street, that you were cured of consumption, and that you were very "sincere in your belief;" but in two months afterward when you were "stone dead" of consumption you forgot to come back and "correct your report;" hence the idea spread like wildfire all over the country, that if a man had a hole in his lungs as big as a barn door, he only need have a gallon or less of nitrate of silver, strong as aquafortis squirted into the cavity, and Richard was himself again. Longer observations proved to Trousseau that he had made a hasty report and the promised second edition of his book, never appeared. The practice was taken up in this country but it has now been almost entirely abandoned because the disposition to hack or hem or clear the throat or to give frequent little dry coughs, called a "hacking cough" almost always arises from the condition of the lungs or the stomach, and a kind of tickling sensation is felt in the throat, referred to the little hollow at the top of the breast bone, but that sensation is induced by the state of the lungs or stomach, a foot or two away as the reader remembers in his own experience on receiving a knock on the elbow, the most disagreeable sensation was felt at the end of the fingers ; when the leg is said to be asleep from sitting in such a position as to arrest the circulation, the ugliest feeling is in the foot, the cause of which is above the knee. When a drop or crumb of bread has " gone the wrong way," an intense and spiteful tickling is excited in the throat, not because the crumb is there, but farther down in the lungs and nature adopts this method of exciting a desire to cough, in the throat, because a cough is the forcible expulsion of air from the bottom of the lungs, intending to carry the offending crumb before it along one of the pipes into the throat and thence outside ; in this case, applying anything to the throat to repress or take away the tickling is depriving nature of her only means of ridding herself of the offending crumb ; in case of consumption, it is the yellow matter in the lungs which makes the throat tickle preparatory to cough ; but removing the tickling by any means only stops the cough, and allows the " phlegm " to remain in the lungs, to accumulate, to fill up the lungs, so that scarcely any air can get into them ; hence, one of the most constant and distressing symptoms of consumption is shortness of breath, caused by the patient taking medicine to repress the cough ; hence it is that all medicines to stop coughs, in colds and consumptions literally kill, instead of cure.

